# Effects of Virtual Reality Therapy Combined With Conventional Rehabilitation on Pain, Kinematic Function, and Disability in Patients With Chronic Neck Pain: Randomized Controlled Trial

**DOI:** 10.2196/42829

**Published:** 2024-04-24

**Authors:** Qifan Guo, Liming Zhang, Leo Lianyi Han, Chenfan Gui, Guanghui Chen, Chunyan Ling, Wei Wang, Qiang Gao

**Affiliations:** 1 West China Hospital Sichuan University Chengdu China; 2 Department of Rehabilitation Medicine West China Hospital Sichuan University Chengdu China; 3 Department of Rehabilitation Medicine The First Affiliated Hospital Sun Yat-Sen University Guangzhou China; 4 Biostatistics Group State Key Laboratory of Genetic Engineering Greater Bay Area Institute of Precision Medicine (Guangzhou), Fudan University Guangzhou China; 5 Department of Traumatology and Orthopedics of Traditional Chinese Medicine The First Affiliated Hospital of Guangxi University of Chinese Medicine Nanning China; 6 Department of Acupuncture and Tuina The First Affiliated Hospital of Guangxi University of Chinese Medicine Nanning China

**Keywords:** virtual reality, neck pain, disability, kinematic function, rehabilitation, physiotherapy, neck, pain, chronic, therapy, kinematic, efficacy, patient, effect

## Abstract

**Background:**

Neck pain is a common condition that leads to neck motor dysfunction and subsequent disability, with a significant global health care burden. As a newly emerging tool, virtual reality (VR) technology has been employed to address pain and reduce disability among patients with neck pain. However, there is still a lack of high-quality studies evaluating the efficacy of VR therapy combined with conventional rehabilitation for patients with chronic neck pain, particularly in terms of kinematic function.

**Objective:**

This study aims to investigate the effect of VR therapy combined with conventional rehabilitation on pain, kinematic function, and disability in patients with chronic neck pain.

**Methods:**

We conducted an assessor-blinded, allocation-concealed randomized controlled trial. Sixty-four participants experiencing chronic neck pain were randomly allocated into the experimental group that underwent VR rehabilitation plus conventional rehabilitation or the control group receiving the same amount of conventional rehabilitation alone for 10 sessions over 4 weeks. Pain intensity, disability, kinematic function (cervical range of motion, proprioception, and mean and peak velocity), degree of satisfaction, and relief of symptoms were evaluated at 3 timepoints (baseline, postintervention, and at 3 months follow-up). A 2*3 mixed repeated measures analysis of variance was utilized for analyzing the difference across indicators, with a significant difference level of .05.

**Results:**

Both groups demonstrated significant improvements in pain, disability, and kinematic functions (*P*<.05) at postintervention and at 3-month follow-up. The experimental group showed superior therapeutic outcomes compared to the control group in pain reduction (mean difference from the baseline: 5.50 vs 1.81 at posttreatment; 5.21 vs 1.91 at the 3-month follow-up, respectively; *P*<.001), disability improvement (mean difference from baseline: 3.04 vs 0.50 at posttreatment; 3.20 vs 0.85 at the 3-month follow-up, respectively; *P*<.001), and enhanced kinematic functions (*P*<.05). Moreover, participants in the experimental group reported better satisfaction and relief of symptoms than the control group (*P*<.05), with better initiative for exercising during the follow-up period. However, there was no between-group difference of improvement in proprioception. No adverse events were reported or observed in our research.

**Conclusions:**

The findings of our study support the efficacy of combining VR therapy with conventional rehabilitation in alleviating pain, enhancing kinematic function, and reducing disability of patients with chronic neck pain. Future research should focus on refining the therapeutic protocols and dosages for VR therapy as well as on optimizing its application in clinical settings for improved convenience and effectiveness.

**Trial Registration:**

Chinese Clinical Trial Registry ChiCTR2000040132; http://www.chictr.org.cn/showproj.aspx?proj=64346

## Introduction

Chronic neck pain is a prevalent global health issue, with reported prevalence rates ranging from 10% to 24% [1,2]. This condition is closely associated with motor dysfunction in the cervical region, characterized by deficits in various kinematic functions of the neck [3-5]. Cervical kinematic functions can be operationally defined as the capacity of the neck muscles to generate and regulate movement of the head and neck, including range of motion (ROM), which is the degree of movement that can be achieved in various directions of the cervical spine, velocity, coordination, strength, and endurance. These parameters can be quantified through specific evaluations and measurements such as ROM assessments, manual muscle testing, and functional movement tests. Prior studies have proven that motor dysfunction occurs commonly in patients with neck pain [6-8], and these dysfunctions are highly correlated with the level of pain and disability. That is because weakness in neck muscle strength and coordination will provide more unstable support of the neck segments and additional stress on the neck structure, which restricts the patient’s neck movement and results in pain [9]. These diminished motor dysfunctions as well as worse pain and disability undoubtedly impair a patient’s work performance and quality of life, leading to large economic losses [10]. Given the abovementioned pivotal role of cervical kinematics in neck pain, interventions aimed at improving motor function hold promise in managing this condition.

To date, active exercise is recommended to be a valid therapy for patients with chronic neck pain based on the current clinical guidelines [11,12]. Virtual reality (VR) is a unique form of exercise established by Morton Heiling in 1962 and has been evolving over the past 60 years [13,14]. VR technology commonly generates virtual environments resembling the real world through devices such as computers or head-mounted displays and interacts with patients to enable them to accomplish the targeted therapeutic goals [15,16]. Regarding the economics of VR treatment, studies [17,18] have reported low costs in VR-based treatments. The hardware devices involved in VR therapy are readily available and inexpensive. Additionally, the one-time cost of patient-specific VR software allows for repeated use, making VR applications relatively less expensive in medical settings. VR serves as a valuable assessment and intervention tool in rehabilitation due to its clinical benefits supported by ongoing research [19], and orthopedic and neurological rehabilitation are the common areas where VR therapy is utilized in clinical practice [20,21]. The potential therapeutic mechanisms of VR include task-oriented repetition, positive feedback, and embodied simulation [22].

As a noninvasive method of pain reduction, VR therapy, both independently and in combination with other interventions, has been investigated in numerous studies. Prior research [23-25] has demonstrated the potential of VR therapy to alleviate pain and disability in patients with orthopedic conditions such as rheumatoid arthritis, shoulder impingement syndrome, and low back pain. However, to date, there is still less research exploring the effects of VR therapy or combined treatment on individuals with chronic neck pain, particularly in terms of improving the cervical motor function [26,27]. Mukherjee et al [28] investigated the efficacy of VR therapy in the treatment of cervical spondylosis. Their findings revealed that patients who underwent VR therapy along with conventional physiotherapy demonstrated notable improvements in pain intensity and active cervical ROM (CROM) compared to those who underwent conventional therapy alone in the short-term period (*P*<.05). However, another study [29] reported that after receiving 4 weeks of VR training, patients with neck pain exhibited significant improvement in mean and maximal velocity, with no observed improvement in CROM indicators compared with the control group. A recent meta-analysis [30] consisting of 2 randomized controlled trials (RCTs) suggested that VR therapy combined with kinematic training could enhance the global perceived effect, patient satisfaction, and general health of patients with neck pain compared to treatment with kinematic training alone. However, evidence supporting the efficacy of VR therapy in strengthening cervical kinematic function remains inconclusive. Given the current gaps in research and the conflicting findings, further high-quality studies are warranted to ascertain the therapeutic effectiveness of VR therapy or combined treatments for individuals with chronic neck pain. Therefore, this RCT aims to evaluate the effects of VR therapy combined with conventional rehabilitation on pain, kinematic functions, and disability in patients with chronic neck pain.

## Methods

### Study Design and Ethics Approval

This study was designed as an assessor-blinded, allocation-concealed RCT (Multimedia Appendix 1). Ethics approval for this study was obtained from the West China Hospital Clinical Trials and Biomedical Ethics Committee of Sichuan University (approval: HX-IRB-AF-18-2021-1102). This trial was registered in the Chinese Clinical Trial Registry (ChiCTR2000040132) on November 22, 2020. This study conformed to the Declaration of Helsinki, and all patients provided written consent after recruitment.

### Participants

This study was conducted at the Department of Rehabilitation Medicine in West China Hospital. Patients were recruited through various channels such as social networks, posters, and brochures from October to December 2021. Inclusion criteria included age of 18 years and older, a diagnosis of chronic neck pain (>3 months) by a physician, reported pain intensity ≥3 on the numeric rating scale (NRS), and disability ≥6 on the neck disability index. Exclusion criteria included existing vestibular pathology, cervical fracture/dislocation, whiplash injuries, neurological/cardiovascular/respiratory disorders affecting patients’ physical performance, inability to provide informed consent, and pregnancy.

### Randomization

Randomization was performed using a computer-generated sequence from Randomization.com, with a researcher not involved in treatment overseeing the process. Patients were allocated to either the experimental group or control group based on the odd or even nature of the assigned number within sealed opaque envelopes to ensure blind allocation. Although a blinded researcher assessed the patients during the trial, blinding was not feasible for participants or therapists due to the layout of the VR therapy.

### Intervention

#### VR Treatment

The VR equipment that we used included several hardware and software (Chengdu Feiming Technology Co, Ltd). Hardware included a Pico G2 4k head-mounted VR glass, monitor screen, and optical motion capture camera. Patients wearing VR glasses sat at a distance of 100 cm from the front of the monitor screen. The monitor will display the real-time virtual images that patients see during the experimental process. Therapists can assess the patient's real-time treatment stage by looking at the monitor screen and provide corresponding assistance. During treatment, the optical motion capture camera and customized software collected and analyzed the cervical movement trajectories. Meanwhile, considering the requirement of fully immersive VR therapy, a specific shoulder strap was designed for patient safety during treatment.

In VR therapy, 3 modules (ROM, proprioception, and velocity modules) were designed to enhance the specific kinematic functions of individuals experiencing chronic neck pain. These modules involved patients engaging in targeted cervical movements to attain therapeutic objectives through visual cues. Prior to the beginning of the treatment, each patient underwent a practice trial to mitigate any potential learning biases. Throughout the VR session, patients were fully immersed in a virtual setting resembling a living room, where they could manipulate virtual objects to interact with designated targets. The VR equipment incorporated visual and auditory feedback to augment the interactive and engaging nature of the therapy. The detailed descriptions of the 3 modules are provided below.

**Figure 1:**
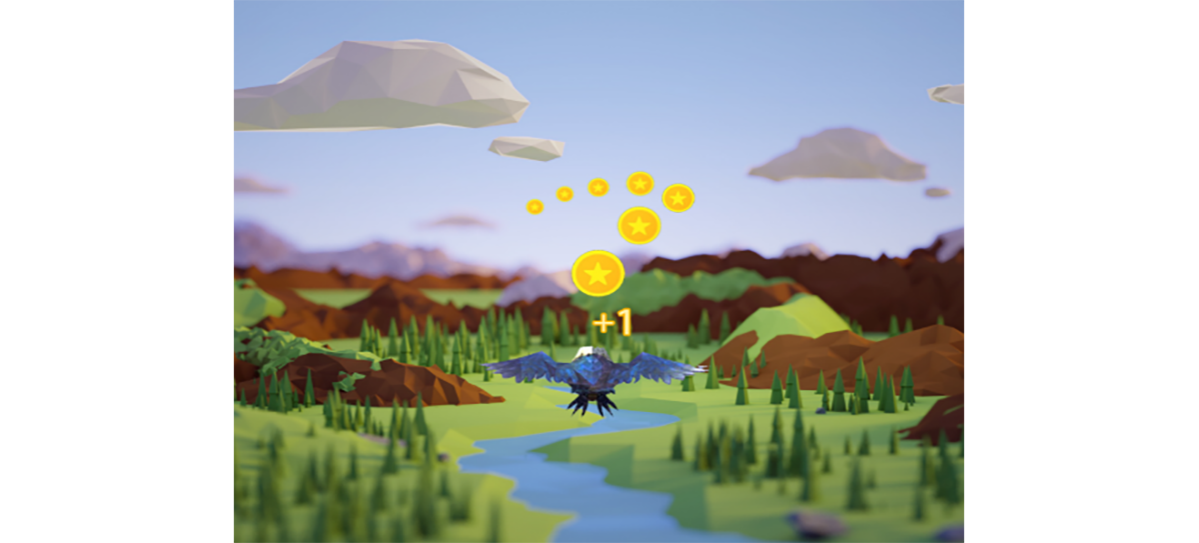
Range of motion module. The bird in the picture is manipulated by the patient’s cervical movement (flexion, extension, lateral flexion, and rotation) to catch the gold and avoid obstacles appearing in all directions.

In the ROM module, a virtual flying bird is manipulated by the patients’ cervical movement. Patients could move birds by neck flexion, extension, lateral flexion, and rotation movement to catch gold or avoid the fire rings appearing in the scene. The placement of the gold items and fire rings was based on baseline kinematic data, with the game’s difficulty adjusted continuously to facilitate gradual improvements in CROM across all movement directions (Figure 1).

**Figure 2:**
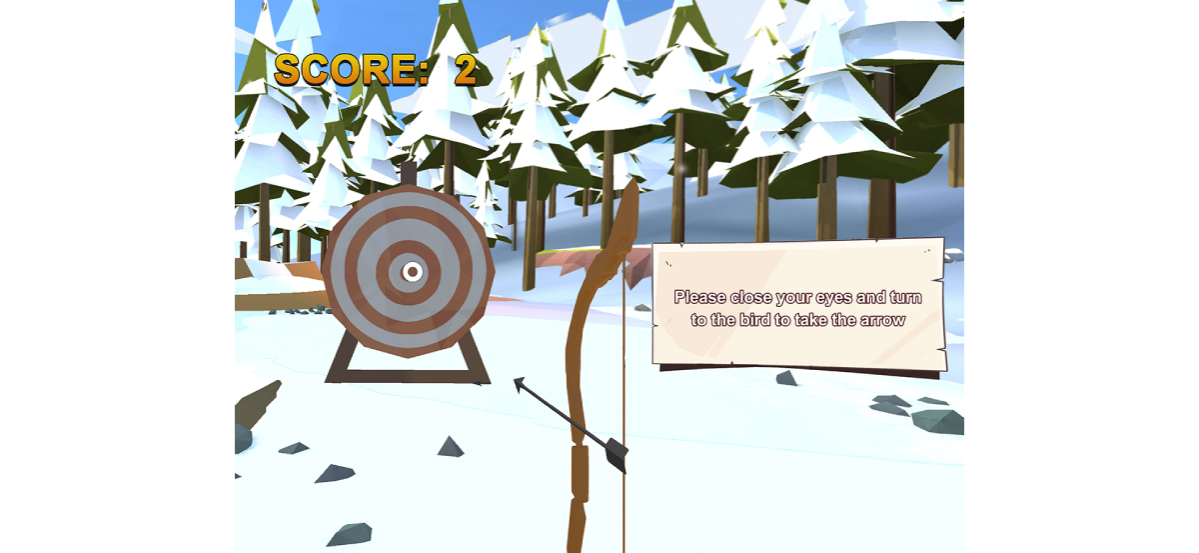
Proprioception module. The patient was asked to remember the initial bull’s-eye position and then close the eyes. Thereafter, the computer would guide the neck of the patient with the eyes closed to a specific position. The patient needs to move the neck back to the original position based on memory and manipulate the bow and arrow to shoot the bull’s-eye.

In the proprioception module, patients engage in tasks requiring them to control a virtual bow and arrow by using cervical movements to aim and shoot at a bull’s-eye target with closed eyes. Initially, patients face the screen to align the arrow with the bull’s-eye, memorizing this starting position. Subsequently, patients close their eyes and follow instructions from the VR system to move their necks to a specific position. They then have to return their neck to the initial position (representing the bull’s-eye location) and shoot the arrow. The relocation error, indicating the angular deviation between the shot point and the bull’s-eye, serves as a measure of patients’ proprioceptive abilities (Figure 2).

**Figure 3:**
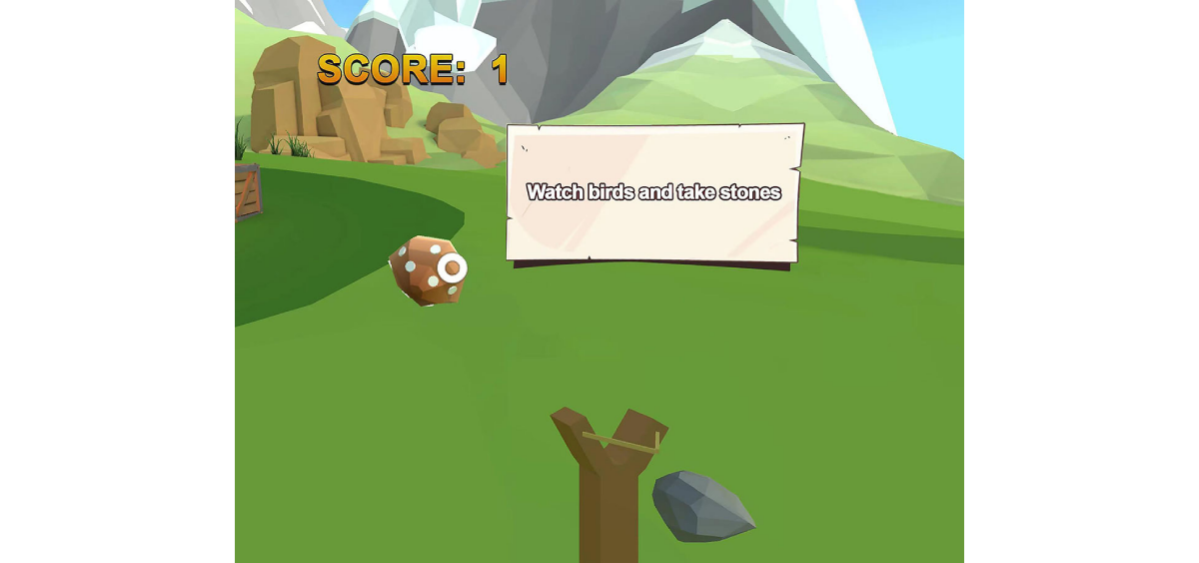
Velocity module. Patients were asked to manipulate stones at maximum neck movement speed to hit randomly appearing virtual mushrooms and obtain the corresponding scores.

In the velocity module, participants were tasked with hitting randomly appearing mushrooms within the virtual scene by manipulating virtual stones with cervical movements. Upon mushroom appearance, patients adjusted the slingshot position by moving their neck and launched a stone to hit the mushroom before it disappeared after 5 seconds. Subsequent mushrooms would appear sequentially, prompting patients to swiftly target and strike them. Patient performance was scored based on the success rate of hitting the mushrooms, thereby fostering patient engagement and compliance with the virtual therapy protocol (Figure 3).

#### Conventional Rehabilitation

Patients in the experimental group received a 10-minute conventional rehabilitation session consisting of 5 minutes of active exercise (eg, muscle stretching exercise, strengthening exercise, sling exercise therapy), supported by established guidelines [11,31]. Additionally, patients underwent a 5-minute transcutaneous electrical nerve stimulation for analgesia, which was validated for efficacy in prior studies [2]. Patients in the control group were treated with 30 minutes of conventional rehabilitation, including 15 minutes of active exercise modalities same as the experimental group and an additional 15 minutes of transcutaneous electrical nerve stimulation therapy. The prescription of conventional rehabilitation was tailored based on each patient’s motor dysfunction and the clinical expertise of the rehabilitation therapists.

### Procedure

Sixty-four patients were randomly allocated into the experimental group or control group, and they underwent 10 treatment sessions over 4 weeks. In the experimental group, interventions included 20 minutes of VR therapy and 10 minutes of conventional rehabilitation per session, while the control group received 30 minutes of only conventional rehabilitation per session. Throughout the treatment, patient safety was closely monitored, and sessions were halted if any adverse events (eg, motion sickness, headache, falls) occurred. Patients were allowed to resume training once the symptoms subsided; otherwise, they were advised to discontinue participation in the study. Following the intervention, both groups were encouraged to continue neck exercises at home for 3 months after treatment. On completion of the follow-up period, each patient was asked to rate their frequency of neck exercise within the 3-month interval to represent their adherence to continued neck exercise. The rating scale ranged from 0 to 4 (0 = no training; 1 = 0-1 hours of training per week; 2 = 1-2 hours of training per week; 3 = 2-3 hours of training per week, ≥4 = >3 hours of training per week). A comparison of the data sets from both groups was conducted to observe the patients’ initiative in training at unsupervised situations.

### Outcome Measures

All outcome measures were evaluated at 3 timepoints: preintervention, immediately postintervention, and 3-month follow-up. The primary outcomes focused on pain and disability (key concerns for individuals seeking medical help for neck pain). These outcomes were evaluated using offline scales. Secondary outcomes included kinematic indicators (eg, CROM, proprioception, mean and peak velocity), patient satisfaction, and relief of symptoms, which are all crucial aspects in the rehabilitation process for chronic neck pain. These secondary outcomes were assessed using a combination of web-based VR equipment and offline scales.

### Primary Outcomes

#### NRS

The NRS was used to measure the current neck pain intensity. NRS graded the pain intensity from 0 (no pain) to 10 (worst pain imaginable), with higher scores indicating worse pain. Pain levels were categorized as mild (1-3), moderate (4-6), and severe (≥7) based on the score range [32-34]. The NRS has shown validity and reliability, with a minimum clinically important difference (MCID) of 2.7 established in previous studies [35,36].

#### Neck Disability Index

The neck disability index was employed as a self-reported questionnaire to measure neck pain–related disability. It consisted of 10 items about activities of daily living, with each item scored from 0 (absence of disability) to 5 (complete disability). The neck disability index is recognized for its validity and reliability, with an MCID of 3.5 points considered significant [37,38].

### Secondary Outcomes

#### CROM

CROM was measured using a VR device in 6 directions: flexion, extension, left and right rotation, and left and right lateral flexion. The results were calculated by taking the average of 3 measuring values. This VR equipment evaluation approach demonstrates high repeatability and sensitivity on these cervical kinematics parameters (ie, CROM, proprioception, mean and peak velocity). The reliability and validity of VR devices to measure CROM have been validated. The minimal detectable change (MDC) of CROM in different directions has been previously reported, while the value changed across the 6 movements ranging from 3.6° to 6.5° [39,40].

#### Proprioception

Proprioception was defined as the perception of change in direction, position, or speed produced by motor organs (eg, muscles, tendons, joints) in 6 directions. It was calculated as the mean of the relocation difference in 3 tests. Prior studies have reported the psychometric properties of VR equipment evaluating proprioception [4,41] but not provided the MCID.

#### Mean and Peak Velocity

Mean and peak velocity are crucial indicators reflecting cervical kinematic functions. The mean and peak velocity in 4 directions (flexion, extension, left and right rotation) were obtained by calculating the average values of 3 assessed data on angular velocity during the trial. VR devices have shown good repeatability in measuring cervical motion velocity. Although the MDC for average speed is 14.31°/s, that for maximum speed is 34.95°/s [4,42].

#### Global Perceived Effect

Global perceived effect is a self-administered questionnaire applied to evaluate patient satisfaction and the relief of symptoms in this study [43]. The satisfaction level ranges from –5 (totally dissatisfied) to 5 (totally satisfied). Similarly, patients could report their relief of symptoms by using the Global Perceived Effect scale, with lower scores representing worse therapeutic effects. These 2 indicators were only measured immediately postintervention and at 3 months after intervention.

### Sample Size Calculation

The NRS was chosen as the primary outcome measure in this study. With reference to a previous study [44], the effect size estimate for the NRS was medium (SE 0.25). The correlation between repeated measures was assumed to be 0.5. Three measurements were presumed to be performed (baseline, postintervention, and 3-month follow-up) with a sphericity correction of 0.5. Based on the statistical power of 0.85 and an α level of .05, a total sample size of 50 patients was initially estimated. To account for potential dropout rates that have been observed to exceed 15% in similar studies [27,29,45], a conservative dropout rate of 25% was chosen to ensure sufficient patients for statistical analysis, resulting in a final inclusion of 64 patients. The sample size calculation was conducted using the G*Power software (version 3.1.7; University of Düsseldorf).

### Statistical Analysis

Statistical analysis was conducted using the SPSS statistical software (version 25.0; IBM Corp) by a blinded researcher. Data analysis followed the intention-to-treat principle, while the Shapiro-Wilk test was applied to check the normality of various data. Descriptive statistics were used to reflect the different types of results such as mean and standard deviation for the parametric variables and median and quartiles for the nonparametric variables. Group equivalence was assessed via the 2-sided independent-sample *t* test or Pearson chi-squared test by comparing the baseline data between the groups. For most variables (all outcomes except the relief of symptoms), which showed normal distribution, a 2*3 mixed repeated measures analysis of variance (ANOVA) with 1 between-subject factor (treatment) and 1 within-subject factor (time) was performed to compare all variables. Post hoc comparisons were conducted using the Bonferroni test, with *P* values for multiple comparisons adjusted using SPSS software. To address violations of the sphericity assumption, the Greenhouse-Geisser correction was applied. With regard to the analysis of the relief of symptoms, nonparametric statistics were used due to the skewed distribution of data. To account for the dropouts, multiple imputations were used to fill the missing data. To show the effect sizes of observed between- or within-group change, partial eta squared and rank correlation were calculated for the parametric and nonparametric variables, respectively. Based on the previous study [46], effect sizes were classified into small (0.2-0.5), medium (0.5-0.8), and large effect sizes (≥0.8). *P* values less than .05 were indicated to be statistically significant.

## Results

### Baseline Measures

**Figure 4:**
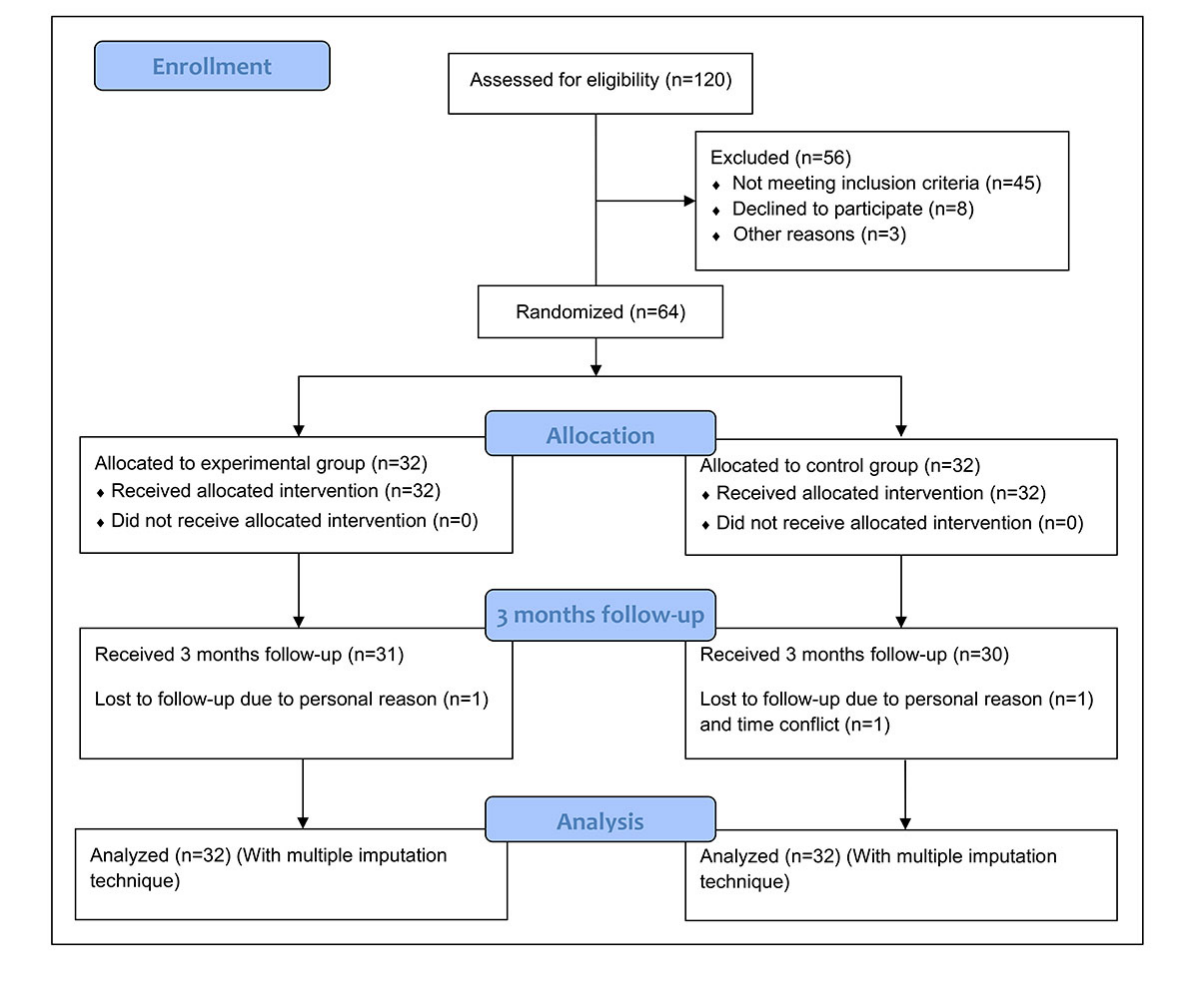
Flow diagram showing participants’ flow and follow-up evaluation.

A total of 120 patients underwent the initial screening for eligibility, of which 56 participants were excluded. Following screening, 64 participants were randomly allocated to either the experimental group or the control group. In the 3-month follow-up period, 3 (5%) participants dropped out of the study due to time conflicts or personal reasons. The flow diagram of participant recruitment and research is shown in Figure 4. The baseline characteristics of the participants in both groups are detailed in Table 1. As shown, there was no between-group difference in age, gender, etiology, disability, pain, and other kinematic indicators. No adverse events were reported during treatment, except some discomfort (eg, complaints of heavy helmets, slightly aggravated pain). No differences existed between the 2 groups over the compliance of the patients continuing neck exercise during the 3-month follow-up period (experimental group 2.31, SD 1.25 vs control group 1.96, SD 1.19; *P*=.22). However, a higher proportion of experimental group participants (16 out of 31) engaged in neck exercises for an average of at least 2 hours per week during the follow-up period compared to control group participants, where only 30% (9/30) achieved this level of compliance, indicating the actual differences between the 2 groups.

**Table 1:** Baseline characteristics of the participants in the experimental and the control groups.

Variables		Experimental group (n=32)	Control group (n=32)	*P* value
Age (years), mean (SD)		35.94 (11.02)	40.09 (11.97)	.15^a^
Gender (female), n (%)		25 (78)	22 (69)	.40^b^
Etiology (idiopathic neck pain), n (%)		16 (50)	21 (66)	.43^b^
Disability (Neck Disability Index), mean (SD)		11.34 (2.74)	10.72 (2.32)	.33^a^
Pain intensity (Numeric Rating Scale), mean (SD)		5.38 (1.39)	5.16 (1.42)	.54^a^
**Range of motion, mean (SD)**				
	Flexion	53.96 (10.69)	51.60 (9.99)	.37^a^
	Extension	63.55 (8.88)	58.42 (12.85)	.07^a^
	Left flexion	37.62 (7.78)	38.42 (8.90)	.71^a^
	Right flexion	38.16 (8.67)	40.91 (8.47)	.20^a^
	Left rotation	71.92 (9.8)	70.21 (8.96)	.47^a^
	Right rotation	71.86 (8.16)	69.43 (9.55)	.28^a^
**Proprioception, mean (SD)**				
	Flexion	2.96 (1.31)	3.33 (1.80)	.36^a^
	Extension	3.03 (1.25)	3.16 (1.65)	.73^a^
	Left flexion	2.85 (1.45)	3.00 (1.29)	.67^a^
	Right flexion	2.73 (1.07)	2.86 (1.57)	.71^a^
	Left rotation	1.96 (0.70)	2.53 (1.36)	.08^a^
	Right rotation	2.85 (1.35)	2.98 (1.64)	.72^a^
**Mean velocity, mean (SD)**				
	Flexion	12.54 (2.80)	11.02 (2.68)	.07^a^
	Extension	14.24 (2.52)	14.90 (2.38)	.28^a^
	Left rotation	15.64 (3.47)	15.15 (3.80)	.59^a^
	Right rotation	17.27 (2.51)	16.59 (2.58)	.29^a^
**Peak velocity, mean (SD)**				
	Flexion	68.63 (17.18)	76.77 (26.13)	.15^a^
	Extension	77.62 (21.63)	77.71 (17.05)	.99^a^
	Left rotation	88.71 (18.46)	88.97 (18.72)	.96^a^
	Right rotation	94.15 (14.24)	100.66 (22.47)	.17^a^

^a^Independent sample *t* test.

^b^Pearson chi-squared test.

### Primary Variables Measure

#### Neck Disability

**Figure 5:**
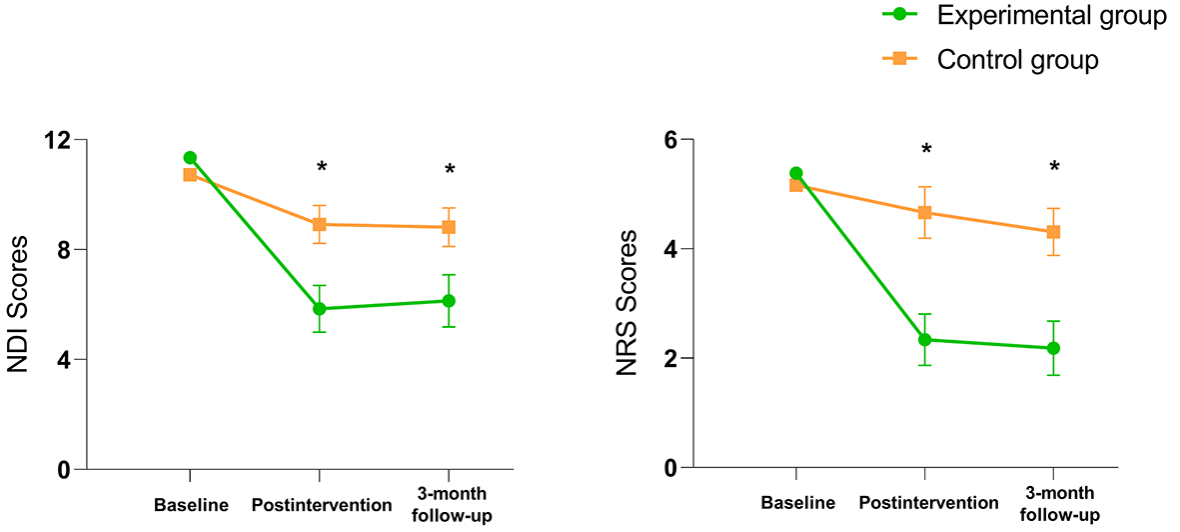
Rehabilitation effect of virtual reality therapy on disability and pain intensity. NDI: neck disability index; NRS: numeric rating scale; *: a statistically significant difference (*P*<0.05) between the two groups at that timepoint (postintervention or 3-month follow-up).

As presented in Table 2 and Figure 5, a repeated measures ANOVA showed a main effect of group (*F*_1,3_=12.738; *P*=.001; η_p_^2^=0.291), time (*F*_2,62_=124.140; *P*<.001; η_p_^2^=0.800), and the group*time interaction (*F*_2,62_=31.620; *P*<.001; η_p_^2^=0.505) on neck disability. Compared with those in the control group, participants in the experimental group showed a significant alleviation in neck disability at postintervention (*P*<.001; η_p_^2^=0.517) and 3-month follow-up (*P*<.001; η_p_^2^=0.438). Furthermore, therapies in both groups were shown to improve disability in patients with chronic neck pain after intervention or 3-month follow-up in comparison with the baseline (*P*<.01). Further, the extent of disability alleviation in the experimental group exceeded the MCID at both measurement timepoints (5.50 at posttreatment; 5.21 at the 3-month follow-up), while the controls showed a reduction in the disability score by 1.81 and 1.91 points compared to baseline. A higher percentage of experimental group participants experienced disability score reductions exceeding the MCID compared to the control group at both timepoints (experimental group: 29/32, 91% vs control group: 9/32, 28% at posttreatment; experimental group: 25/31, 81% vs control group 6/30, 20% at the 3-month follow-up).

**Table 2:** Within- and between-group differences in outcome measures.

Variables		Preintervention, mean (SD)			Postintervention, mean (SD)			Cohen *d*^a^		3-month follow-up, mean (SD)			Cohen *d*		Group*time, *F* test *(df)*
		Experimental group	Control group	Experimental group		Control group			Experimental group		Control group				
Disability		11.34 (2.74)	10.72 (2.32)	5.84 (2.38)^b,c^		8.91 (1.92)^c^	0.517		6.13 (2.66)^b,c^		8.81 (1.96)^b^	0.438		31.62 (2, 62)^d^	
Pain intensity		5.38 (1.39)	5.16 (1.42)	2.34 (1.31)^b,c^		4.66 (1.31)	0.582		2.18 (1.38)^b,c^		4.31 (1.20)	0.587		27.28 (2, 62)^d^	
**Range of motion**															
	Flexion	53.96 (10.69)	51.60 (9.99)	64.78 (9.01)^b,c^		54.25 (8.25)	0.582		67.96 (6.03)^b,c^		53.76 (8.82)	0.680		11.31 (1.592, 49.363)^d^	
	Extension	63.55 (8.88)	58.42 (12.85)	70.89 (7.07)^b,c^		59.78 (9.93)	0.395		69.09 (8.97)^b,c^		54.29 (8.48)	0.603		8.74 (2, 62)^d^	
	Left flexion	37.62 (7.78)	38.42 (8.90)	44.23 (7.37)^b,c^		38.11 (8.22)	0.277		47.02 (4.02)^b,c^		35.83 (8.69)	0.578		22.40 (2, 62)^d^	
	Right flexion	38.16 (8.67)	40.91 (8.47)	45.78 (7.43)^b,c^		40.18 (7.55)	0.192		45.42 (5.73)^b,c^		35.17 (6.50)^b^	0.514		22.48 (2, 62)^d^	
	Left rotation	71.92 (9.8)	70.21 (8.96)	79.85 (6.47)^b,c^		70.85 (10.09)	0.305		84.92 (5.39)^b,c^		67.86 (11.13)	0.660		21.97 (2, 62)^b^	
	Right rotation	71.86 (8.16)	69.43 (9.55)	77.19 (7.34)^b,c^		70.62 (8.10)	0.239		82.23 (7.52)^b,c^		65.77 (6.83)	0.682		26.72 (2, 62)^d^	
**Proprioception**															
	Flexion	2.96 (1.31)	3.33 (1.80)	2.55 (1.28)		2.71 (1.38)	0.007		1.66 (1.02)		1.55 (0.81)	0.007		0.58 (2, 62)	
	Extension	3.03 (1.25)	3.16 (1.65)	2.43 (0.86)		2.67 (1.03)	0.035		2.53 (1.40)		2.24 (0.70)	0.026		0.96 (2, 62)	
	Left flexion	2.85 (1.45)	3.00 (1.29)	2.57 (1.13)		2.52 (1.38)	0.001		2.02 (1.28)		2.41 (1.02)	0.064		0.62 (2, 62)	
	Right flexion	2.73 (1.07)	2.86 (1.57)	2.28 (0.82)		2.98 (1.12)	0.182		2.56 (1.36)		2.66 (1.08)	0.003		1.33 (2, 62)	
	Left rotation	1.96 (0.70)	2.53 (1.36)	2.61 (1.38)		2.21 (0.92)	0.053		2.04 (1.31)		2.79 (0.97)	0.209		6.28 (1.687, 52.289)^d^	
	Right rotation	2.85 (1.35)	2.98 (1.64)	2.36 (1.19)		2.29 (1.43)	0.002		1.81 (0.95)		2.47 (1.45)	0.120		1.25 (2, 62)	
**Mean velocity**															
	Flexion	12.54 (2.80)	11.02 (2.68)	15.71 (2.74)		12.93 (3.12)	0.270		14.30 (2.59)		12.58 (2.27)	0.218		1.28 (2, 62)	
	Extension	14.24 (2.52)	14.90 (2.38)	14.85 (2.00)		13.68 (2.97)	0.085		15.22 (1.66)^b,c^		13.14 (2.39)	0.337		6.53 (2, 62)^d^	
	Left rotation	15.64 (3.47)	15.15 (3.80)	18.13 (3.76)^b^		16.44 (3.28)	0.105		20.27 (3.46)^b,c^		16.99 (2.13)^b^	0.452		3.80 (1.609, 19.872)^e^	
	Right rotation	17.27 (2.51)	16.59 (2.58)	18.75 (2.12)		17.56 (1.93)	0.156		17.50 (2.03)		16.63 (2.02)	0.079		0.24 (2, 62)	
**Peak velocity**															
	Flexion	68.63 (17.18)	76.77 (26.13)	81.74 (20.69)^b^		72.61 (21.01)	0.092		117.76 (31.10)^b,c^		91.55 (21.72)^b^	0.376		14.69 (2, 62)^d^	
	Extension	77.62 (21.63)	77.71 (17.05)	76.17 (16.90)		71.71 (18.45)	0.049		99.55 (25.10)^b,c^		70.74 (14.55)	0.530		13.31 (2, 62)^d^	
	Left rotation	88.71 (18.46)	88.97 (18.72)	93.97 (12.52)		98.71 (27.52)	0.022		173.00 (51.51)^b,c^		132.43 (29.69)^b^	0.365		13.75 (1.522, 47.174)^d^	
	Right rotation	94.15 (14.24)	100.66 (22.47)	102.32 (24.37)		89.02 (13.23)^b^	0.167		124.33 (29.63)^b,c^		93.26 (10.63)	0.534		13.29 (2, 62)^d^	
Satisfaction		N/A^f^	N/A	3.03 (1.33)		1.66 (1.82)	0.860		2.97 (1.28)		1.81 (1.99)	0.693		N/A	
Relief of symptoms		N/A	N/A	3.00 (2.00)		2.00 (3.00)	N/A		4.00 (1.00)		2.00 (2.00)	N/A		N/A	

^a^Cohen *d* was calculated for the differences between postintervention or 3-month follow-up and preintervention in the experimental group compared to the control group.

^b^Indicates *P*<.02 (0.05/3) for within-group comparisons by Bonferroni correction compared to the baseline.

^c^*P*<.05 statistically significant differences were found compared to the control group at the same measuring timepoint.

^d^*P*<0.01 significant main effects were revealed on the group*time interaction.

^e^*P*<.05 significant main effects were revealed on the group*time interaction.

^f^N/A: not applicable.

#### Neck Pain Intensity

For pain, ANOVA results revealed significant differences over time (*F*_1.744,54.077_=87.369; *P*<.001; η_p_^2^=0.738), group (*F*_1,31_=28.138; *P*<.001; η_p_^2^=0.476), and the group*time interaction (*F*_2,62_=27.277; *P*<.001; η_p_^2^=0.468). The post hoc analysis indicated a significant between-group difference at postintervention (*P*<.001; η_p_^2^=0.582) and 3 months postintervention (*P*<.001; η_p_^2^=0.587), with the experimental group representing better enhancement. Compared with baseline, patients in the experimental group experienced pain relief immediately postintervention and at 3-month follow-up, while control group participants did not exhibit significant pain reduction throughout the study. Besides, pain intensity scores decreased in both groups compared to baseline (experimental group 3.04 vs control group 0.50 at posttreatment; experimental group 3.20 vs control group 0.85 at the 3-month follow-up), with patients in the experimental group exceeding the MCID at 2 timepoints. The percentage of data exceeding the MCID significantly differed between the 2 groups (experimental group: 21/32, 66% vs control group: 3/32, 9% at posttreatment; experimental group: 20/31, 65% vs control group 4/30, 13% at the 3-month follow-up).

### Secondary Variables Measure

#### CROM

The results of ANOVA on CROM revealed a significant effect of the group, time, and group*time interaction (*P*<.05). Participants in the experimental group obtained greater ROM improvement in 6 directions at postintervention and at 3-month follow-up (*P*<.05) compared to the control group participants. Notably, significant changes were observed after intervention and follow-up in the experimental group from those at baseline (*P*<.05), while no differences were observed in the control group. The experimental group participants achieved ROM improvements exceeding the MDC in all directions at both timepoints, except for extension ROM at the 3-month follow-up, highlighting the clinical effectiveness of the intervention in the experimental group compared to the control group. Specific data on this indicator can be found in Multimedia Appendix 2.

#### Proprioception

Regarding proprioception, the ANOVA results revealed no significant difference in the group*time interaction for 6 directions, except for left rotation. For the proprioception of left rotation, significant effects occurred in the group*time interaction. The post hoc analysis showed that patients receiving VR intervention attained lesser improvement after the 3-month follow-up than the control group. However, no within-group differences were reported. Upon further analysis of other directions, proprioception in flexion, extension, and right rotation directions was found to achieve improvement in both groups after treatment and follow-up versus the baseline, and proprioception of left flexion showed a noticeable improvement after the follow-up in comparison with the baseline. However, the between-group analysis showed no marked difference in all directions. The detailed data regarding this parameter are presented in Multimedia Appendix 2.

#### Mean and Peak Velocity

There were significant main effects for the interaction between time and group (*P*<.05) for the mean velocity of extension and left rotation. The post hoc analysis revealed significant gains after the 3-month follow-up in the experimental group compared to that in the control group or baseline for these 2 directions. No significant effects for the interaction between time and group were found in the mean velocity of flexion and right rotation. For intergroup comparisons, patients receiving VR training showed better improvement in the mean velocity of flexion than the control group, which was not found in the right rotation direction. As for the within-group comparison, both groups showed superiority over baseline in the mean velocity of flexion at posttreatment and the 3-month follow-up, and the mean velocity of right rotation remained negative. However, the magnitude of improvement in the mean velocity in both the groups did not surpass the corresponding MDC in any direction. A repeated measures ANOVA showed a main effect for the group*time interaction for the peak velocity in all 4 directions. Compared with the baseline, the experimental group participants gained significant enhancement (*P*<.05) at the 3-month follow-up. Furthermore, the between-group comparisons supported better therapeutic effects with VR devices than the control after intervention and 3-month follow-up period (Table 2). Increased maximal velocity of flexion and left rotation directions was significantly higher in the experimental group over MDC after the follow-up, which did not occur in the other directions or the control group. For specific data on the mean and peak velocity, please refer to Multimedia Appendix 2.

#### Global Perceived Effect

Considering patient satisfaction, the results revealed a significant between-group difference at postintervention and 3-month follow-up (experimental group 3.03, SD 1.33 vs control group 1.66, SD 1.82) with an advantage to the experimental group. The within-group analysis showed no significant effects among different timepoints for the 2 groups. In the Mann-Whitney *U* test, obvious between-group differences were found in the relief of symptoms at postintervention (experimental group 3.00, SD 2.00 vs control group 2.00, SD 3.00) and 3-month follow-up (experimental group 4.00, SD 1.00 vs control group 2.00, SD 2.00). Furthermore, significant improvements were observed in the experimental group after the 3-month follow-up compared to those at postintervention, while no differences were observed in the control group (Table 2). The specific data related to these indicators can be obtained in Multimedia Appendix 2.

## Discussion

### Overview

This RCT was intended to compare the benefits of combining VR therapy and conventional rehabilitation with those of conventional rehabilitation alone for treating chronic neck pain. Overall, our results show that patients in both groups reported reduced pain and disability and demonstrated improved kinematic functions. Direct comparisons between the 2 groups revealed that VR treatment in addition to conventional rehabilitation was superior to conventional rehabilitation alone for improvement in the pain, disability, and kinematic indicators, and the effects of combined therapy could be maintained over the 3-month follow-up period. Additionally, participants in the VR therapy group reported higher satisfaction levels, better symptom improvement, and greater willingness to engage in exercises during the follow-up period.

### Effects of VR Therapy Combined With Conventional Rehabilitation on Pain and Disability

Although reduced pain and disability were found in both treatment groups at 2 timepoints, these indicators were decreased nearly 3 times more in the experimental group than in the control group. Furthermore, the improvement of pain and disability observed at 2 measuring timepoints in the experimental group was higher than the MCID, which has been previously reported as 2.7 and 3.5 points, respectively [37,38]. This finding indicates the significant and clinical effectiveness of VR therapy in addition to conventional rehabilitation in alleviating pain and disability. The corresponding size effects were medium, highlighting the notable differences between the 2 groups.

Multiple studies have shown that patients with neck pain experienced significant improvements in pain intensity with VR treatment compared to baseline [45,47] and markedly superior to control groups receiving laser training [27] or conventional rehabilitation [28]. Further, a recent meta-analysis [48] incorporating 8 RCTs revealed that better analgesic effects were found in the multimodal intervention (VR technique in combination with other therapies) than in the other interventions and in the patients treated in the clinic or research unit than the controls. This also provides a new perspective on VR analgesia research. However, some studies have reported conflicting results. For instance, a study [44] investigating the efficacy of a 120-minute VR therapy session for patients with chronic neck pain indicated remarkable improvement in pain intensity at rest or during motion compared to baseline as well as alleviation in the disability level. However, no significant between-group differences were observed in these metrics in the VR intervention group as compared to the 2 control groups undergoing conventional rehabilitation alone or general sensorimotor training plus conventional rehabilitation. This discrepancy may be attributed to the smaller sample size in that study (17 individuals per group) [44], which lowered the statistical power representation of between-group differences. Similarly, the VR gaming scenario utilized in that study [44] lacked sufficient visual and auditory feedback compared to the VR design in our research, and this might have limited the analgesic effect of VR treatment.

The potential efficacy of VR therapy in reducing neck pain and disability may be attributed to its ability to enhance coordination between the deep and superficial cervical muscles [49]. Poor sensorimotor control by cervical muscles in patients with neck pain has been indicated in previous research [5,9] and is considered to trigger associated disability and kinematic disorders. Although muscle activation was not evaluated in this study, VR therapy appears to promote the function and coordination of cervical muscles, thereby reducing the stress on cervical segments and alleviating neck pain and disability. Another possible reason could be the deep engagement required by the virtual environment, blocking the transmission of sensory information related to pain and achieving analgesic effects.

### Effects of VR Therapy Combined With Conventional Rehabilitation on Cervical Kinematic Function

The secondary outcomes yielded interesting findings that VR therapy could increase the ROM, mean velocity, and peak velocity at 2 timepoints compared to those in the baseline or control group. This conclusion was consistent with that reported in previous research [28,44,50]. Tejera et al [50] in 2021 reported the positive results of VR therapy on increasing CROM in patients with chronic neck pain, which can be attributed to the sufficient feedback provided by VR devices. The visualization of images was widely perceived as useful in activating the corticospinal system and enhancing the intensity of muscle recruitment, thereby improving the overall neck kinematic functions. Fowler et al [51] showed that VR might encourage patients to turn their heads farther and faster by its effect on reducing fear of movement, which has been reported in other studies [52-54]. Besides these, continuous progressive VR treatment dosage based on real-time assessment data on motor function assisted patients in restoring their motor function.

As can be observed from the mean and maximum velocity data in various directions, the experimental group always showed no between-group differences after training but showed between-group differences after the 3 months follow-up compared to the control group, suggesting that this may be attributed to the insufficient training time during the intervention. Upon analyzing the training length after the intervention, we could see that the training frequency of patients in the experimental group was higher than that of the control group during the follow-up period, reaching an average of 1-2 hours of training per week, and more training time outside of the experiment would probably promote further improvement of motor function. These findings indicate that researchers as well as clinical specialists should pay more attention to the supervision and education of home-based active exercise in the future.

Regarding proprioception, both groups showed significant improvement in several directions after treatment or 3-month follow-up; however, no between-group differences were found. Prior studies [49,55] have confirmed that multiple exercise programs, including head relocation practice, gaze stability, eye-follow, and eye/head coordination, are effective in improving proprioception. In this study, however, only head relocation practice was used (the participant was instructed to memorize the head-neck position and try to find the initial position with eyes closed after moving), and satisfactory results were obtained. Moreover, other studies [27,44] have utilized alternative proprioceptive training with similarly favorable outcomes. Some investigators noticed that eye-follow and eye/head coordination training greatly enhanced patients’ accuracy, which was likely attributed to improved motor control and coordination of the neck [47]. This suggests that more consideration should be given to focus on all forms of proprioceptive training in the clinical management of patients with chronic neck pain.

### Effects of VR Therapy Combined With Conventional Rehabilitation on Satisfaction and Relief of Symptoms

Besides the indicators mentioned above, the marked between-group difference was observed in patient satisfaction and relief of symptoms at both timepoints, with some advantages in the combined treatment. These 2 self-rating indicators are considered important for recovering from chronic neck pain. The enjoyment derived from VR equipment, multiple visual and auditory feedback, personalized tasks, and adjustable difficulty levels likely contributed to the higher satisfaction levels and greater therapeutic efficacy in the experimental group. Notably, no adverse events such as motion sickness were reported during the research period.

### Limitations

Several limitations of this study warrant consideration. The absence of a placebo group receiving sham VR therapy raises concerns about the potential overestimation of VR therapy’s therapeutic effects. However, the substantial improvements in pain, disability, and CROM surpassing the MCID suggest that the effect may be due to the treatment itself other than the placebo effect. Moreover, the impact of varying durations of conventional rehabilitation (30 minutes vs 10 minutes) on therapeutic outcomes remains uncertain, potentially influencing the perceived efficacy of VR treatment. Additionally, the inability to blind patients due to the experimental nature of this study introduces a risk of bias. The lack of long-term follow-up data further limits the generalizability of the findings. Lastly, the absence of assessment indicators for mental function and quality of life hinders the comprehensive evaluation of VR therapy’s overall therapeutic efficacy.

### Implications

This study provides support for the effectiveness of a combined approach involving VR therapy and conventional rehabilitation in managing chronic neck pain. However, uncertainties persist regarding the optimal dosage, underlying mechanisms of VR therapy, and the comparative effectiveness of different VR equipment types (eg, semi-immersive, nonimmersive). Future investigations should design specific trials to address these knowledge gaps. Furthermore, exploring the synergistic benefits of integrating VR training with other evidence-based interventions such as manipulation and sensorimotor training is warranted.

### Conclusions

In conclusion, the integration of VR intervention with conventional rehabilitation demonstrates significant improvements in pain, disability, and kinematic function among patients with chronic neck pain at both postintervention and 3-month follow-up assessments. Although patients can benefit from conventional rehabilitation alone, the combination of VR therapy and conventional rehabilitation is more effective for improvement in the abovementioned indicators. Considering the higher satisfaction as well as greater training initiative in the experimental group and the absence of adverse events, this feasible and effective intervention could be integrated into the standard rehabilitation treatment plan for patients with chronic neck pain. Future research endeavors should focus on refining therapeutic regimens, determining optimal dosages for VR therapy, and streamlining the implementation of this intervention in clinical settings to enhance convenience and efficacy.

## Appendix

Multimedia Appendix 1 CONSORT-EHEALTH (Consolidated Standards of Reporting Trials of Electronic and Mobile Health Applications and Online TeleHealth) checklist (v 1.6.1).

Multimedia Appendix 2 The main and simple effect data sets.

## Abbreviations

ANOVA: analysis of variance
CROM: cervical range of motion
MCID: minimum clinically important difference
MDC: minimal detectable change
NRS: numeric rating scale
RCT: randomized controlled trial
ROM: range of motion
VR: virtual reality

## References

[1] Cohen SP (2015). Epidemiology, diagnosis, and treatment of neck pain. Mayo Clin Proc.

[2] Martimbianco ALC, Porfírio Gustavo JM, Pacheco RL, Torloni MR, Riera R (2019). Transcutaneous electrical nerve stimulation (TENS) for chronic neck pain. Cochrane Database Syst Rev.

[3] Stanton TR, Leake HB, Chalmers KJ, Moseley GL (2016). Evidence of impaired proprioception in chronic, idiopathic neck pain: systematic review and meta-analysis. Phys Ther.

[4] Sarig Bahat H, Chen X, Reznik D, Kodesh E, Treleaven J (2015). Interactive cervical motion kinematics: sensitivity, specificity and clinically significant values for identifying kinematic impairments in patients with chronic neck pain. Man Ther.

[5] Röijezon Ulrik, Björklund Martin, Bergenheim M, Djupsjöbacka Mats (2008). A novel method for neck coordination exercise--a pilot study on persons with chronic non-specific neck pain. J Neuroeng Rehabil.

[6] Rudolfsson T, Björklund Martin, Djupsjöbacka Mats (2012). Range of motion in the upper and lower cervical spine in people with chronic neck pain. Man Ther.

[7] Lee H, Nicholson LL, Adams RD (2005). Neck muscle endurance, self-report, and range of motion data from subjects with treated and untreated neck pain. J Manipulative Physiol Ther.

[8] Beltran-Alacreu H, López-de-Uralde-Villanueva Ibai, Calvo-Lobo C, Fernández-Carnero Josué, La Touche R (2018). Clinical features of patients with chronic non-specific neck pain per disability level: A novel observational study. Rev Assoc Med Bras (1992).

[9] Harel NY, Song K, Tang X, Strittmatter SM (2010). Nogo receptor deletion and multimodal exercise improve distinct aspects of recovery in cervical spinal cord injury. J Neurotrauma.

[10] Takasaki H, Treleaven J, Johnston V, Jull G (2013). Contributions of physical and cognitive impairments to self-reported driving difficulty in chronic whiplash-associated disorders. Spine (Phila Pa 1976).

[11] Blanpied PR, Gross AR, Elliott JM, Devaney LL, Clewley D, Walton DM, Sparks C, Robertson EK (2017). Neck Pain: Revision 2017. J Orthop Sports Phys Ther.

[12] Wang xueqiang, Wang Yuling, Zhang Zhijie (2020). Exercise therapy for neck pain: Chinese expert consensus. Journal of Shanghai University of Sport.

[13] Sarig Bahat H, Weiss PL, Laufer Y (2010). The effect of neck pain on cervical kinematics, as assessed in a virtual environment. Arch Phys Med Rehabil.

[14] Iskander M, Ogunsola T, Ramachandran R, McGowan R, Al-Aswad LA (2021). Virtual reality and augmented reality in ophthalmology: a contemporary prospective. Asia Pac J Ophthalmol (Phila).

[15] Lopez-de-Uralde-Villanueva I, Beltran-Alacreu H, Fernandez-Carnero J, Kindelan-Calvo P, La Touche R (2016). Widespread pressure pain hyperalgesia in chronic nonspecific neck pain with neuropathic features: a descriptive cross-sectional study. Pain Physician.

[16] Muñoz-García Daniel, López-de-Uralde-Villanueva Ibai, Beltrán-Alacreu Héctor, La Touche R, Fernández-Carnero Josué (2017). Patients with concomitant chronic neck pain and myofascial pain in masticatory muscles have more widespread pain and distal hyperalgesia than patients with only chronic neck pain. Pain Med.

[17] Cipresso P, Giglioli IAC, Raya MA, Riva G (2018). The past, present, and future of virtual and augmented reality research: a network and cluster analysis of the literature. Front Psychol.

[18] Brown A, Green T (2016). Virtua reality: low-cost tools and resources for the classroom. TechTrends.

[19] Pawassar CM, Tiberius V (2021). Virtual reality in health care: bibliometric analysis. JMIR Serious Games.

[20] Wu J, Zeng A, Chen Z, Wei Y, Huang K, Chen J, Ren Z (2021). Effects of virtual reality training on upper limb function and balance in stroke patients: systematic review and meta-meta-analysis. J Med Internet Res.

[21] Gumaa M, Rehan Youssef A (2019). Is virtual reality effective in orthopedic rehabilitation? a systematic review and meta-analysis. Phys Ther.

[22] Gray ML, Goldrich DY, McKee S, Schaberg M, Del Signore A, Govindaraj S, Iloreta AM (2021). Virtual reality as distraction analgesia for office-based procedures: a randomized crossover-controlled trial. Otolaryngol Head Neck Surg.

[23] Lin D, Lin Y, Chai H, Han Y, Jan M (2007). Comparison of proprioceptive functions between computerized proprioception facilitation exercise and closed kinetic chain exercise in patients with knee osteoarthritis. Clin Rheumatol.

[24] Pekyavas NO, Ergun N (2017). Comparison of virtual reality exergaming and home exercise programs in patients with subacromial impingement syndrome and scapular dyskinesis: Short term effect. Acta Orthop Traumatol Turc.

[25] Matheve T, Bogaerts K, Timmermans A (2020). Virtual reality distraction induces hypoalgesia in patients with chronic low back pain: a randomized controlled trial. J Neuroeng Rehabil.

[26] Sarig Bahat Hilla, Takasaki H, Chen X, Bet-Or Y, Treleaven J (2015). Cervical kinematic training with and without interactive VR training for chronic neck pain - a randomized clinical trial. Man Ther.

[27] Sarig Bahat H, Croft K, Carter C, Hoddinott A, Sprecher E, Treleaven J (2018). Remote kinematic training for patients with chronic neck pain: a randomised controlled trial. Eur Spine J.

[28] Mukherjee M, Bedekar N, Sancheti P, Shyam A (2020). Immediate and short-term effect of virtual reality training on pain, range of motion, and kinesiophobia in patients with cervical spondylosis. Indian J Phys Ther Res.

[29] Bahat HS, German D, Palomo G, Gold H, Nir YF (2020). Self-kinematic training for flight-associated neck pain: a randomized controlled trial. Aerosp Med Hum Perform.

[30] Ahern MM, Dean LV, Stoddard CC, Agrawal A, Kim K, Cook CE, Narciso Garcia A (2020). The effectiveness of virtual reality in patients with spinal pain: a systematic review and meta-analysis. Pain Pract.

[31] Zronek M, Sanker H, Newcomb J, Donaldson M (2016). The influence of home exercise programs for patients with non-specific or specific neck pain: a systematic review of the literature. J Man Manip Ther.

[32] Williamson A, Hoggart B (2005). Pain: a review of three commonly used pain rating scales. J Clin Nurs.

[33] Wan Li, Zhao Qing, Chen Jun (2020). Expert consensus on the application of pain evaluation questionnaires in China(2020). Chinese Journal Of Painology.

[34] No authors listed (1994). Pain: clinical manual for nursing practice. Nurs Stand.

[35] Juul T, Søgaard Karen, Davis AM, Roos EM (2016). Psychometric properties of the Neck OutcOme Score, Neck Disability Index, and Short Form-36 were evaluated in patients with neck pain. J Clin Epidemiol.

[36] Young IA, Dunning J, Butts R, Mourad F, Cleland JA (2019). Reliability, construct validity, and responsiveness of the neck disability index and numeric pain rating scale in patients with mechanical neck pain without upper extremity symptoms. Physiother Theory Pract.

[37] Cleland JA, Childs JD, Fritz JM, Whitman JM (2006). Interrater reliability of the history and physical examination in patients with mechanical neck pain. Arch Phys Med Rehabil.

[38] Pool JJM, Ostelo RWJG, Hoving JL, Bouter LM, de Vet HCW (2007). Minimal clinically important change of the Neck Disability Index and the Numerical Rating Scale for patients with neck pain. Spine (Phila Pa 1976).

[39] Sarig-Bahat H, Weiss PL, Laufer Y (2009). Cervical motion assessment using virtual reality. Spine (Phila Pa 1976).

[40] Audette I, Dumas J, Côté Julie N, De Serres SJ (2010). Validity and between-day reliability of the cervical range of motion (CROM) device. J Orthop Sports Phys Ther.

[41] Sarig Bahat H, Watt P, Rhodes M, Hadar D, Treleaven J (2020). High-vs. low-tech cervical movement sense measurement in individuals with neck pain. Musculoskelet Sci Pract.

[42] Sarig Bahat H, Sprecher E, Sela I, Treleaven J (2016). Neck motion kinematics: an inter-tester reliability study using an interactive neck VR assessment in asymptomatic individuals. Eur Spine J.

[43] Evans R, Bronfort G, Maiers M, Schulz C, Hartvigsen J (2014). "I know it's changed": a mixed-methods study of the meaning of Global Perceived Effect in chronic neck pain patients. Eur Spine J.

[44] Nusser M, Knapp S, Kramer M, Krischak G (2021). Effects of virtual reality-based neck-specific sensorimotor training in patients with chronic neck pain: A randomized controlled pilot trial. J Rehabil Med.

[45] Cetin H, Kose N, Oge HK (2022). Virtual reality and motor control exercises to treat chronic neck pain: A randomized controlled trial. Musculoskelet Sci Pract.

[46] Cohen J (1988). Statistical Power Analysis for the Behavioral Sciences.

[47] Rezaei I, Razeghi M, Ebrahimi S, et al (2019). A novel virtual reality technique (Cervigame®) compared to conventional proprioceptive training to treat neck pain: a randomized controlled trial. J Biomed Phys Eng.

[48] Guo Q, Zhang L, Gui C, Chen G, Chen Y, Tan H, Su W, Zhang R, Gao Q (2023). Virtual reality intervention for patients with neck pain: systematic review and meta-analysis of randomized controlled trials. J Med Internet Res.

[49] Jull G, Falla D, Treleaven J, Hodges P, Vicenzino B (2007). Retraining cervical joint position sense: the effect of two exercise regimes. J Orthop Res.

[50] Tejera DM, Beltran-Alacreu H, Cano-de-la-Cuerda R, Leon Hernández JV, Martín-Pintado-Zugasti A, Calvo-Lobo C, Gil-Martínez A, Fernández-Carnero J (2020). Effects of virtual reality versus exercise on pain, functional, somatosensory and psychosocial outcomes in patients with non-specific chronic neck pain: a randomized clinical trial. Int J Environ Res Public Health.

[51] Fowler CA, Ballistrea LM, Mazzone KE, Martin AM, Kaplan H, Kip KE, Murphy JL, Winkler SL (2019). A virtual reality intervention for fear of movement for veterans with chronic pain: protocol for a feasibility study. Pilot Feasibility Stud.

[52] López-de-Uralde-Villanueva Ibai, Notario-Pérez Ricardo, Del Corral T, Ramos-Díaz Bernardo, Acuyo-Osorio M, La Touche R (2017). Functional limitations and associated psychological factors in military personnel with chronic nonspecific neck pain with higher levels of kinesiophobia. Work.

[53] Harvie DS, Smith RT, Moseley GL, Meulders A, Michiels B, Sterling M (2020). Illusion-enhanced virtual reality exercise for neck pain: a replicated single case series. Clin J Pain.

[54] Yilmaz Yelvar Gul Deniz, Çırak Yasemin, Dalkılınç Murat, Parlak Demir Yasemin, Guner Z, Boydak A (2017). Is physiotherapy integrated virtual walking effective on pain, function, and kinesiophobia in patients with non-specific low-back pain? Randomised controlled trial. Eur Spine J.

[55] Revel M, Minguet M, Gregoy P, Vaillant J, Manuel JL (1994). Changes in cervicocephalic kinesthesia after a proprioceptive rehabilitation program in patients with neck pain: a randomized controlled study. Arch Phys Med Rehabil.

