# Supplementary material for: Effects of Virtual Reality Therapy Combined With Conventional Rehabilitation on Pain, Kinematic Function, and Disability in Patients With Chronic Neck Pain: Randomized Controlled Trial
Source: JMIR Serious Games. 2024 Apr 24;12:e42829. doi: 10.2196/42829 (PMC11079768; doi:10.2196/42829)
Supplement: Multimedia Appendix 2 [file games_v12i1e42829_app2.docx]

***Primary variables measure***

1. ***Neck disability***

*A repeated measure ANOVA showed a main effect of group (F_(1, 31)_=12.738, P<0.01, ηp^2^=0.291), time (F_(2, 62)_=124.140, P<0.01, ηp^2^=0.800), and the group by time interaction (F_(2, 62)_=31.620, P<0.01, ηp^2^=0.505).*

1. ***Neck pain intensity***

*ANOVA results revealed significant differences over time (F_(1.744, 54.077)_=87.369, P<0.01, ηp^2^=0.738), group (F_(1, 31)_=28.138, P<0.01, ηp^2^=0.476), and the group*time interaction (F_(2, 62)_=27.277, P<0.01, ηp^2^=0.468).*

***Secondary variables measure***

1. ***CROM***

***1.1 Flexion***

*ANOVA results revealed significant differences over time (F_(1.640, 50.826)_=25.869, P<0.01, ηp^2^=0.455), group (F_(1, 31)_=34.573, P<0.01, ηp^2^=0.527), and the group*time interaction (F_(1.592, 49.363)_=11.308, P<0.01, ηp^2^=0.267).*

***1.2 Extension***

*ANOVA results revealed significant differences over time (F_(1.563, 48.447)_=6.895, P<0.01, ηp^2^=0.182), group (F_(1, 31)_=22.964, P<0.01, ηp^2^=0.426), and the group*time interaction (F_(2, 62)_=8.736, P<0.01, ηp^2^=0.220).*

***1.3 Left Flexion***

*ANOVA results revealed significant differences over time (F_(2, 62)_=7.630, P<0.01, ηp^2^=0.198), group (F_(1, 31)_=13.663, P<0.01, ηp^2^=0.306), and the group*time interaction (F_(2, 62)_=22.398, P<0.01, ηp^2^=0.419).*

***1.4 Right Flexion***

*ANOVA results revealed significant differences over time (F_(2, 62)_=7.649, P<0.01, ηp^2^=0.198), group (F_(1, 31)_=7.022, P<0.01, ηp^2^=0.185), and the group*time interaction (F_(2, 62)_=22.478, P<0.01, ηp^2^=0.42).*

***1.5 Left Rotation***

*ANOVA results revealed significant differences over time (F_(1.657, 51.376)_=12.076, P<0.01, ηp^2^=0.280), group (F_(1, 31)_=23.804, P<0.01, ηp^2^=0.434), and the group*time interaction (F_(2, 62)_=21.968, P<0.01, ηp^2^=0.415).*

***1.6 Right Rotation***

*ANOVA results revealed significant differences over time (F_(1.601, 49.622)_=6.191, P<0.01, ηp^2^=0.166), group (F_(1, 31)_=21.099, P<0.01, ηp^2^=0.405), and the group*time interaction (F_(2, 62)_=26.715, P<0.01, ηp^2^=0.463).*

1. ***Proprioception***

***2.1 Flexion***

*ANOVA results revealed significant differences over time (F_(2, 62)_=29.557, P<0.01, ηp^2^=0.488), group (F_(1, 31)_=0.396, P=0.534, ηp^2^=0.013), and the group*time interaction (F_(2, 62)_=0.580, P=0.563, ηp^2^=0.018).*

***2.2 Extension***

*ANOVA results revealed significant differences over time (F_(1.690, 52.405)_=8.941, P<0.01, ηp^2^=0.224), group (F_(1, 31)_=0.016, P=0.899, ηp^2^=0.001), and the group*time interaction (F_(2, 62)_=0.964, P=0.387, ηp^2^=0.030).*

***2.3 Left Flexion***

*ANOVA results revealed significant differences over time (F_(1.637, 50.747)_=5.917, P<0.01, ηp^2^=0.160), group (F_(1, 31)_=0.503, P=0.483, ηp^2^=0.016), and the group*time interaction (F_(2, 62)_=0.622, P=0.540, ηp^2^=0.020).*

***2.4 Right Flexion***

*ANOVA results revealed significant differences over time (F_(2, 62)_=0.737, P=0.483, ηp^2^=0.023), group (F_(1, 31)_=1.977, P=0.170, ηp^2^=0.060), and the group*time interaction (F_(2, 62)_=1.331, P=0.272, ηp^2^=0.041).*

***2.5 Left Rotation***

*ANOVA results revealed significant differences over time (F_(2, 62)_=0.448, P=0.637, ηp^2^=0.014), group (F_(1, 31)_=3.183, P=0.084, ηp^2^=0.093), and the group*time interaction (F_(1.687, 52.289)_=6.282, P<0.01, ηp^2^=0.169).*

***2.6 Right Rotation***

*ANOVA results revealed significant differences over time (F_(2, 62)_=5.977, P<0.01, ηp^2^=0.162), group (F_(1, 31)_=1.186, P=0.284, ηp^2^=0.037), and the group*time interaction (F_(2, 62)_=1.248, P=0.294, ηp^2^=0.039).*

1. ***Mean velocity***

***3.1 Flexion***

*ANOVA results revealed significant differences over time (F_(2, 62)_=27.042, P<0.01, ηp^2^=0.466), group (F_(1, 31)_=14.883, P<0.01, ηp^2^=0.324), and the group*time interaction (F_(2, 62)_=1.280, P=0.285, ηp^2^=0.040).*

***3.2 Extension***

*ANOVA results revealed significant differences over time (F_(2, 62)_=0.610, P=0.610, ηp^2^=0.019), group (F_(1, 31)_=4.266, P<0.05, ηp^2^=0.121), and the group*time interaction (F_(2, 62)_=6.533, P<0.01, ηp^2^=0.174).*

***3.3 Left Rotation***

*ANOVA results revealed significant differences over time (F_(2, 62)_=21.405, P<0.01, ηp^2^=0.109), group (F_(1, 31)_=10.231, P<0.01, ηp^2^=0.248), and the group*time interaction (F_(1.609, 19.872)_=3.802, P<0.05, ηp^2^=0.109).*

***3.4 Right Rotation***

*ANOVA results revealed significant differences over time (F_(2, 62)_=8.484, P<0.01, ηp^2^=0.215), group (F_(1, 31)_=6.171, P<0.02, ηp^2^=0.166), and the group*time interaction (F_(2, 62)_=0.235, P=0.791, ηp^2^=0.008).*

1. ***Peak velocity***

***4.1 Flexion***

*ANOVA results revealed significant differences over time (F_(2, 62)_=50.324, P<0.01, ηp^2^=0.619), group (F_(1, 31)_=5.265, P<0.03, ηp^2^=0.145), and the group*time interaction (F_(2, 62)_=14.691, P<0.01, ηp^2^=0.322).*

***4.2 Extension***

*ANOVA results revealed significant differences over time (F_(1.665, 51.623)_=4.862, P<0.05, ηp^2^=0.136), group (F_(1, 31)_=23.040, P<0.01, ηp^2^=0.426), and the group*time interaction (F_(2, 62)_=13.307, P<0.01, ηp^2^=0.300).*

***4.3 Left Rotation***

*ANOVA results revealed significant differences over time (F_(1.302, 40.367)_=103.092, P<0.01, ηp^2^=0.769), group (F_(1, 31)_=7.570, P<0.05, ηp^2^=0.196), and the group*time interaction (F_(1.522, 47.174)_=13.746, P<0.01, ηp^2^=0.307).*

***4.4 Right Rotation***

*ANOVA results revealed significant differences over time (F_(1.580, 48.977)_=10.440, P<0.01, ηp^2^=0.252), group (F_(1, 31)_=16.115, P<0.02, ηp^2^=0.342), and the group*time interaction (F_(2, 62)_=13.285, P<0.01, ηp^2^=0.300).*
